# Novel habitat causes a shift to diurnal activity in a nocturnal species

**DOI:** 10.1038/s41598-018-36384-2

**Published:** 2019-01-18

**Authors:** J. Sean Doody, Colin R. McHenry, David Rhind, Simon Clulow

**Affiliations:** 1 Department of Biological Sciences, University of South Florida – St. Petersburg, St. Petersburg, Florida 33701 USA; 2 School of Environmental and Life Sciences, University of Newcastle, Callaghan, New South Wales 2308 Australia; 3 School of Engineering, University of Newcastle, Callaghan, New South Wales 2308 Australia; 4 School of Biological Sciences, Monash University, Clayton, Victoria 3800 Australia; 5 Department of Biological Sciences, Macquarie University, Sydney, New South Wales 2109 Australia

## Abstract

Plastic responses may allow individuals to survive and reproduce in novel environments, and can facilitate the establishment of viable populations. But can novel environments reveal plasticity by causing a shift in a behavior as fundamental and conspicuous as daily activity? We studied daily activity times near the invasion front of the cane toad (*Rhinella marina*), an invasive species that has colonized much of northern Australia. Cane toads in Australia are nocturnal, probably because diurnal activity would subject them to intolerably hot and dry conditions in the tropical savannah during the dry season. Our study can demonstrate, however, that upon reaching novel environments some toad populations became diurnal. Sandstone gorges offered cane toads novel, deeply shaded habitat. Gorges with an east-west axis (day-long northern shadow), narrow gorges and narrow sections of gorges contained toads that were primarily diurnal, while gorges with a north-south axis, wide gorges and wide sections of gorges contained mainly nocturnal toads. For example, remote camera data (1314 observations of toad activity times over 789 trap days) revealed strictly nocturnal activity at four ‘exposed’ sites (99% of 144 observations over 179 days), compared to mostly diurnal activity at a ‘shaded’ site (78% of 254 observations). Visual encounter surveys confirmed that diurnal activity occurred exclusively at shaded sites, while most nocturnal activity occurred at exposed sites. The close proximity of diurnal and nocturnal toads (4–7 km) provided compelling evidence for the abovementioned physical factors as the proximate cause of the behavioral dichotomy, and for a novel (deeply shaded gorges) environment causing the shift to diurnal activity.

## Introduction

A pivotal response of individual animals to a novel environment is behavioral. Plastic behavioral responses can facilitate the establishment of viable populations in novel environments by allowing individuals to survive and reproduce^1^. Such plasticity is not uncommon - frequent behavioral responses to human-induced changed conditions, for example, include alterations in habitat choice, movements, foraging, social behavior and reproductive behavior (reviewed in^2^). But can novel environments reveal plasticity by causing a shift in a behavior as fundamental and conspicuous as timing of daily activity? An inversion of activity times is not commonly described, despite the fact that it can easily be discerned^3^.

Time is an adaptive behavioral resource: animals maintain daily activity patterns that presumably optimize fitness^4,5^. Although temporal partitioning between competitors and between predators and their prey is a significant mechanism of coexistence in some ecological communities, relatively few animal species are known to invert their activity patterns into the opposite activity phase as a result of predation or competition^3,6^. Physiological constraints such as circadian rhythms as well as behavioral and environmental factors may explain why phase shifts in activity are rare^7–9^.

Despite their apparent rarity, temporal phase shifts in activity patterns have been demonstrated in vertebrates. For example, Norway rats shifted their behavior from nocturnal to diurnal in response to predation by foxes^10^, and the presence of competitors (polecats and otters) caused mink to shift from predominantly nocturnal to mainly diurnal^11^. Abiotic factors can also cause a phase shift in activity; four species of normally diurnal lizards phase shifted to moonlit nights in an open marsh edge, but not in an adjacent forested area where moonlight did not penetrate the forest floor^12^. Finally, life history strategy and physiological condition influenced the likelihood of phase shifts in Salmon^13,14^.

Invasive species are good candidates to search for temporal phase shifts because they often spread into new geographic areas with different selective regimes, manifested in novel predators, competitors or physical environments^15^. Given the serious problems caused by biological invasions, an improved understanding of how behavior contributes to the competitive ability and spread of invasive animals is urgently needed^16^. The cane toad (*Rhinella marina* = ‘*Bufo marinus*’) has invaded over 50 countries from its native range in South and Central America^17^. Its success as an invader is due to its toxic skin that kills predators^18–22^, but it is also a dietary and habitat generalist with high fecundity (reviewed in^17^). Adult cane toads are chiefly nocturnal in both their native and introduced range, although crepuscular activity is not uncommon^17^.

Surprisingly, recent research demonstrated that cane toads near the invasion front ‘phase shifted’ by hydrating during the day in an arid climate^23^. During the dry season in semi-desert Australia, adult cane toads visited reservoirs to rehydrate mainly during daylight hours, in contrast with nocturnal activity exhibited by adult toads in their native geographical range and in more mesic parts of Australia. Diurnal rehydration in reservoirs may have reduced desiccation or thermal stress that would occur in terrestrial retreat sites^23^. Could cane toads also shift their timing of daily activity (e.g., foraging) in a similar way? If so, how could they accomplish this given that they don’t feed on aquatic prey, and given the hot arid tropical savanna that encompasses most of their distribution?

We tested the hypothesis that cane toads have shifted their timing of activity, including feeding, from nocturnal to diurnal near the invasion front, because they encountered a novel environment: sandstone gorges with deeply shaded habitat. We quantified activity times using remote cameras at eight locations in four populations of toads, and we dissected toads to determine if active toads were feeding. We also used visual encounter surveys at six sites to determine (1) the relationship between activity time (diurnal vs. nocturnal) and solar exposure (exposed vs. shaded); and (2) the effect of activity time (diurnal vs. nocturnal) on toad escape responses when approached by observers. We discuss proximate and ultimate explanations for the diversity of toad activity and behavior within such a small geographic area.

## Materials and Methods

We studied timing of activity in the cane toad (*Rhinella marina*) at El Questro Wilderness Park, in the Kimberley region of Western Australia (15°53′42.12′′S, 128°7′56.84′′E) during 2013–2015. The area is in the wet-dry tropics and receives ~800 mm of rainfall annually, with almost no rainfall occurring during the dry season between June and October (Australian Bureau of Meteorology). The area is dominated by savannah woodland and is dissected by sandstone gorges that flow into the Pentecost and King Rivers. The invasive cane toad (*Rhinella marina*) arrived at the sites in 2012–2013 from the east. It has moved incrementally westward across northern Australia since its introduction in Cairns in 1935^17^.

**Figure 1:**
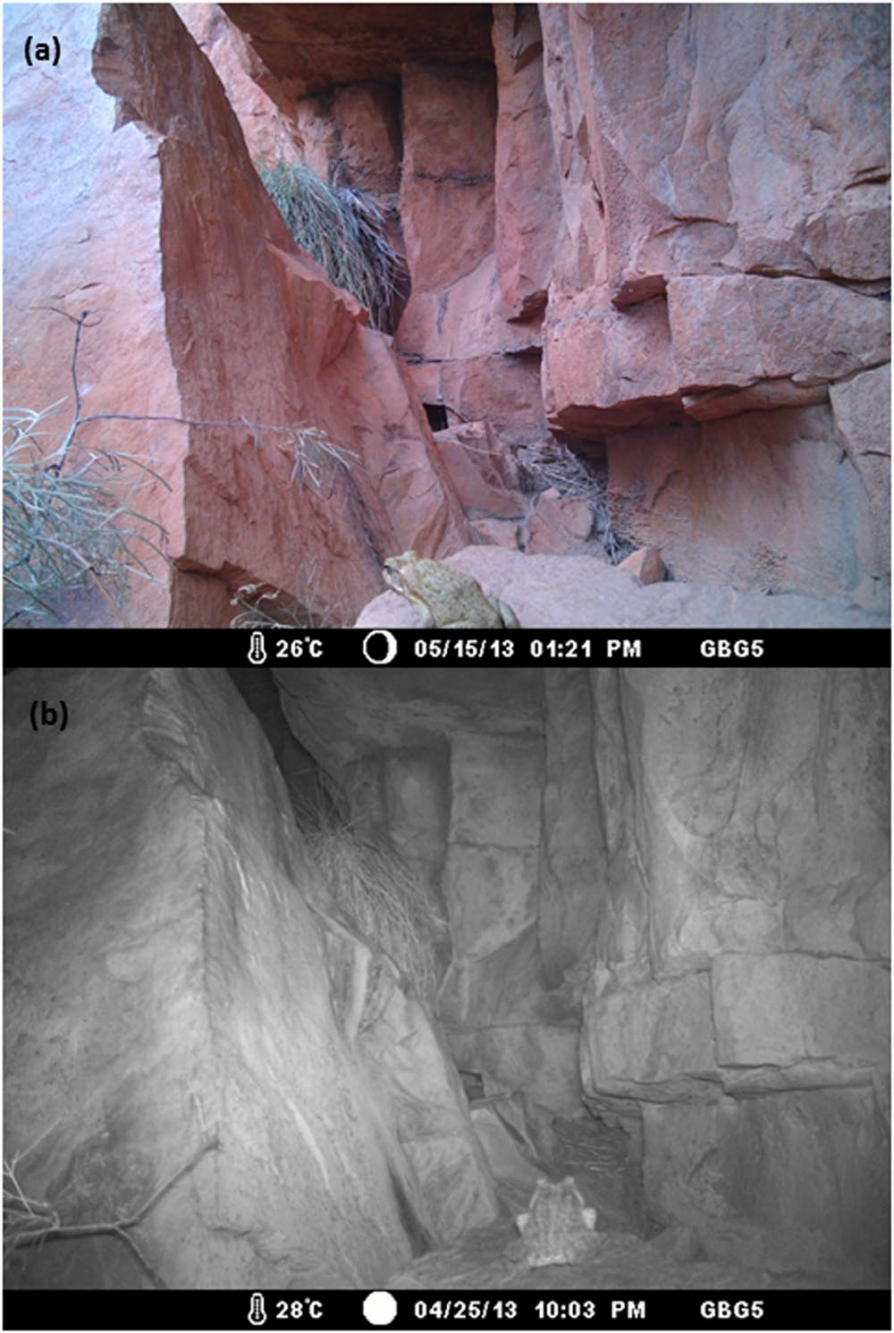
Photographs of adult cane toads (*Rhinella marina*) from a remote camera at Ghost Bat Gorge, El Questro Wilderness Park, Western Australia. (**a**) Diurnal activity; (**b**) nocturnal activity.

Toad activity was quantified using remote game cameras (Moultrie Infra-red I-40®) in 2013–2014 and using visual encounter surveys (VES) in 2014–15. Camera setup and duration differed among sites due to logistical constraints and different objectives in a concurrent study. At Emma Gorge and Ghost Bat Gorge we used 10 and five cameras, respectively, with an inter-camera distance of 100–300 m. At Saddleback and Branco’s Hole we used five cameras with an inter-camera distance of 30–50 m. Cameras were employed for 42–299 days at Emma Gorge, 269–367 days at Ghost Bat Gorge (depending on battery life), and 16 days at Saddleback and Branco’s Hole. The sampling period was primarily the dry season (beginning in May–June), but three cameras continued photographing toads into the wet season. Cameras were secured to trees or rocks and were generally set along rock walls (in gorges) or along the river’s edge to increase the probability of toads crossing near the cameras (Fig. 1). Although the cameras were invariably employed in shade, the larger area around the camera varied from shaded to exposed; for example, exposed sites were dominated by direct sunlight during the day and shaded sites were shaded for most of the day by steep gorge walls. For VES, we conducted 19 surveys ranging from 100–6400 m in length, at six different sites (five gorges). The gorges/sites were chosen to cover a range of exposures, widths and axis orientations (east-west vs. north-south). There were 1–6 surveys per site (mean = 3.2 surveys/site). Each survey lasted 1–7 hours (mean = 2.7 hrs) and involved counting toads conducted during the day or at night by the same observer. We determined the impact of activity (diurnal vs. nocturnal) on escape behavior by scoring each toad’s response to the observer’s approach to 0.3 m away from the toad. Responses were scored as dichotomous: retreating vs. remaining still.

To determine if toads were feeding during the day we dissected samples of toads from Emma Gorge and Ghost Bat Gorge in the late afternoon, but well before dusk. Thus, any stomach contents found would have been ingested during the day. Toads were collected by hand near the Plunge Pool at Emma Gorge and near a spring at Ghost Bat Gorge. Toads were killed by freezing, and were dissected after thawing, within 24 hours of capture. The stomachs were opened to search for prey remains. Although we did not quantify prey remains (in terms of relative abundance, % occurrence by volume, etc.), we documented their occurrence (e.g., ants, beetles, wasps).

All experiments were approved by the University of Newcastle Animal Care and Ethics Committee (approval A-2012-214) and were performed in accordance with the relevant guidelines and regulations.

### Data Analysis

For camera data, the daily count data were summarized into 12, two hour blocks across the 24 hour day for each camera within each site. To compare daily activity patterns between sites and between cameras within sites (for Emma Gorge and Ghost Bat Gorge, which both had more than one camera) we ran a series of generalized linear models with Poisson distribution and a log-link function in JMP (version 11). To investigate differences between daily activity patterns between sites we ran a full factorial model of site by time for all data combined whereby time was treated categorically in the two hour blocks across the day. To investigate difference in daily activity patterns between cameras within each site we ran a full factorial model of camera by time for each individual site. To correct for different numbers of days that each camera recorded activity, an offset (log of number of days recording) was used in the model. Overdispersion was checked and, when found to be significant, an overdispersion correction was applied by using a multiplicative factor to increase the variance expected from the Poisson distribution by the overdispersion observed. When all cameras were modeled together a large amount of overdispersion was observed (the variance was 47.6 times greater than that expected from a Poisson distribution) and adjusting for it led to all effects becoming non-significant. This overdispersion was attributable to one camera that recorded an order of magnitude more toads than the other cameras in the nocturnal periods. To correct for this we ran the site by time and camera by time models with this camera removed. This resulted in significant interaction terms between daily activity patterns and sites with overdispersion reduced to 2.9.

To investigate seasonal effects of activity patterns between Emma Gorge and Ghost Bat Gorge we ran a generalized linear model for the full factorial of site by date using the genmod procedure with a Poisson distribution and log link function in SAS (version 9.4), with a Pearson scale adjustment for overdispersion (Pearson χ^2^/DF).

For VES data, we used a Contingency analysis (Yates χ^24^) to determine if activity (day vs. night) was independent of exposure (exposed vs. shaded) across the sites. To determine the influence of activity times (day vs. night) on the propensity to flee (retreat vs. remain still) we used a Chi-square analysis.

## Results

**Figure 2:**
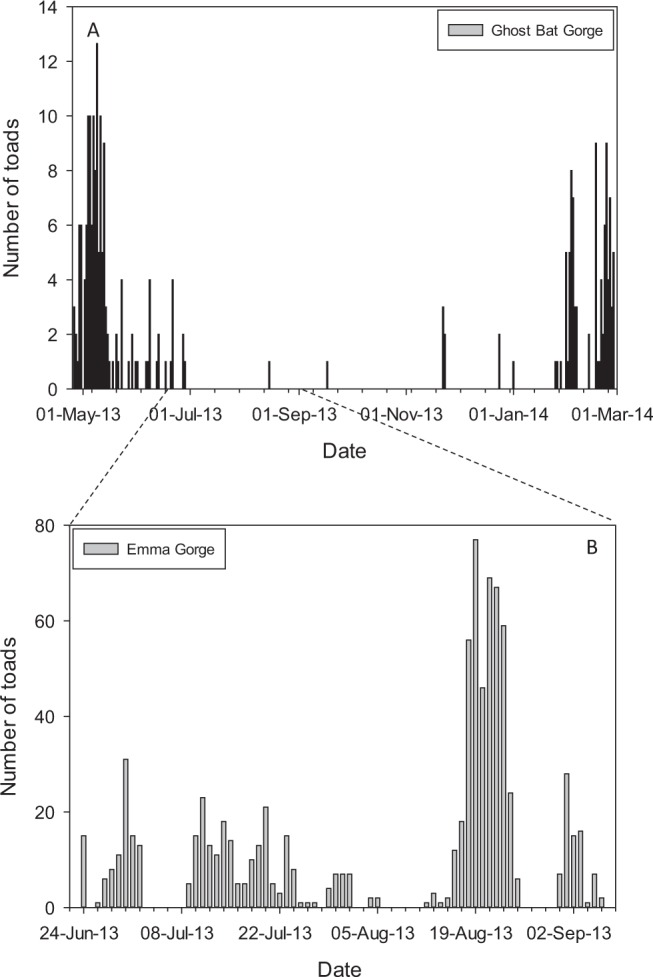
Seasonal variation in adult *R. marina* activity gleaned from remote cameras at two gorges 7 km apart from one another. (**A**) Ghost Bat Gorge camera data (N = 281 observations from two locations), showing peak activity in the late wet season and early dry season and almost no activity during the mid- to late-dry season; (**B**) Emma Gorge camera data (N = 824 observations from one location), showing activity throughout the dry season.

Eight of our cameras photographed enough toads to provide useful data (14–851 toads per camera) (Fig. 1, Table 1). These cameras photographed 1,308 toad encounters over 789 trap days from four populations (Table 1). One camera at Ghost Bat Gorge photographed toads for 10 months, sufficient for determining seasonal variation in activity; toad activity at this site peaked in the late wet season-early dry season (Fig. 2A). When compared to Emma Gorge, there was a significant interaction of site by date for toad activity across the season between the two gorges (F_1,7589_ = 102.5; P < 0.001; Fig. 2). Almost no toad activity occurred at Ghost Bat Gorge in the mid- to late-dry season (mid-July to mid-November) (Fig. 2A). In contrast, although wet season data are lacking, cameras at Emma Gorge captured considerable activity during the mid-dry season (Fig. 2B).

**Table 1:** Activity pattern data for adult *R. marina*, gleaned from remote motion-sensitive cameras.

Population	Site	Habitat	Start date	End date	# days	# obs	# nocturnal	# diurnal
Ghost Bat Gorge	1	shaded	25 Apr 2013	18 Feb 2014	299	254	55	199
Ghost Bat Gorge	2	shaded	25 Apr 2013	6 Jun 2013	42	33	24	9
Emma Gorge	1	both	22 Jun 2013	6 Sep 2013	92	851	821	30
Emma Gorge	2	both	22 Jun 2013	16 Dec 2013	177	35	26	9
Emma Gorge	3	exposed	22 Jun 2013	1 Nov 2013	131	21	21	0
Saddleback	1	exposed	11 Jun 2014	27 Jun 2014	16	36	34	2
Saddleback	2	exposed	11 Jun 2014	27 Jun 2014	16	14	14	0
Branco’s Hole	1	exposed	13 Jun 2014	29 Jun 2014	16	70	70	0
Totals	**8**	—	—	—	**789**	**1314**	**1065**	**249**

For habitat, ‘shaded’ = fully shaded throughout the day due to both the proximity to gorge walls and overstory; ‘exposed’ = camera itself in shaded vegetation but area of view either in sun or surrounded by sun for much of the day.

**Figure 3:**
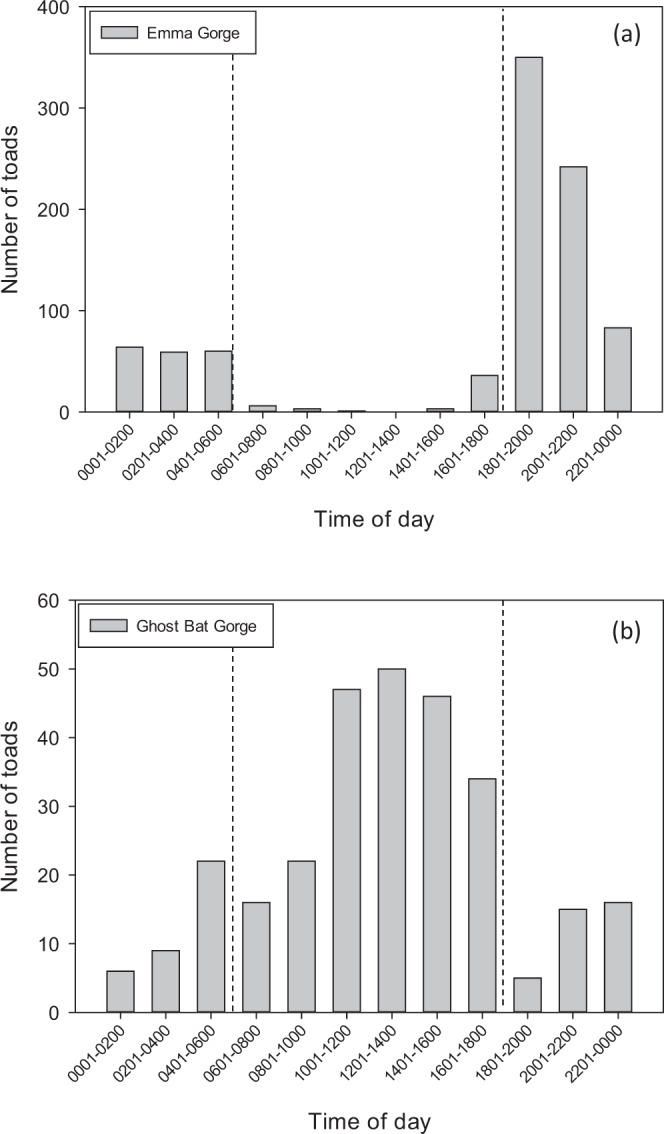
Daily timing of activity of *R. marina* populations from (**a**) Emma Gorge and (**b**) Ghost Bat Gorge. Data are from 1188 photographs taken by remote game cameras (Emma Gorge: 907 observations from three locations; Ghost Bat Gorge: 281 observations from two locations). Dashed lines within panels demarcate day and night. Dashed lines between panels matches the temporal data windows of the two gorges.

Daily timing of activity exhibited marked site variation. The full analysis of activity resulted in significant interaction terms between daily activity patterns across the 24 hour day/night period and sites (LRT χ^2^_(22)_ = 60.9, P < 0.001: Fig. 3). This significant interaction could arise from differences in either diurnal or nocturnal activity patterns, or a combination of both. To better understand where the significant interaction in the daily activity patterns arose, we ran additional models of diurnal activity only by site, and nocturnal activity only by site, for all sites together and for the two sites that displayed nocturnal activity alone (Emma Gorge and Saddleback). The full model for diurnal activity at all sites returned a significant interaction of site by diurnal activity (LRT χ^2^_(10)_ = 30.3, P < 0.001) whereas the model for Emma and Saddleback alone did not (LRT χ^2^ = 1.1, P = 0.95). The model for nocturnal activity at all sites returned a non-significant interaction of site by nocturnal activity (LRT χ^2^_(10)_ = 12.4, P = 0.26). Thus, the interaction in the full model of daily activity patterns by site is explained by the differences in diurnal activity at Ghost Bat Gorge; toads were primarily nocturnal at Emma Gorge, but were mainly diurnal at Ghost Bat Gorge (Fig. 3).

Camera data also indicated no difference in activity patterns among cameras at Emma Gorge. There was some support for differences between cameras at Ghost Bat Gorge (LRT χ^2^_(11)_ = 47.7; P < 0.001); however, this difference was without overdispersion adjustment, which could not be estimated for this subset of the data. Thus, this result should be considered as tentative.

**Figure 4:**
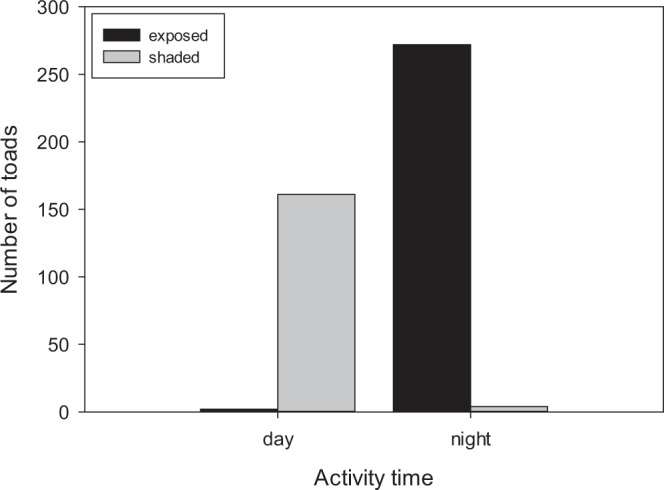
The relationship between solar exposure (exposed vs. shaded) and activity times (diurnal vs. nocturnal) in *R. marina*. Data are from 19 VES surveys across six sites (see Table 2).

According to our VES data, a contingency analysis demonstrated that activity time (day vs. night) was not independent of exposure (exposed vs. shaded) (Yates X^2^ = 422.2, df = 1, p < 0.0001). Diurnal activity occurred exclusively at shaded sites/gorges, while most nocturnal activity occurred at exposed sites/gorges (Table 2; Fig. 4).

**Table 2:** Activity times and flight behavior of 435 cane toads (*Rhinella marina*) at six sites (gorges) with different physical attributes. For gorge width W = wide, N = narrow. For gorge axis N-S = north-south, E-W = east-west. # fleeing = upon approach by the observer to 0.3 m away. EG = Emma Gorge, EPP = Emma Gorge Plunge Pool, GBG = Ghost Bat Gorge, AG = Amalia Gorge, ELG = El Questro Gorge, MG = Moonshine Gorge.

Site	Survey date	Survey time	Day/night	Transect length (km)	Gorge width	Gorge axis	Exposure	# toads	## fleeing (%)
EG	29 May 14	1930–2130	N	1.0	W	N-S	exposed	48	5 (10%)
EG	13 May 15	1930–2100	N	1.0	W	N-S	exposed	64	7 (11%)
EG	8 May 15	1730–1900	N	1.0	W	N-S	exposed	52	6 (12%)
EG	4 May 15	1730–1900	N	1.0	W	N-S	exposed	59	10 (17%)
EG	12 May 15	1000–1400	D	1.0	W	N-S	exposed	0	n/a
EG	6 May 15	1930–2100	N	1.0	W	N-S	exposed	0	n/a
EPP	30 May 14	930–1130	D	0.1	N	N-S	shaded	28	26 (93%)
EPP	4 May 15	1500–1600	D	0.1	N	N-S	shaded	22	17 (77%)
EPP	6 May 15	2100–2200	N	0.1	N	N-S	shaded	2	0 (0%)
GBG	18 May 14	1400–1600	D	0.4	W	E-W	shaded	21	21 (100%)
GBG	23 May 14	1100–1600	D	0.4	W	E-W	shaded	22	22 (100%)
AG	4 May 15	1800–2100	N	1.6	N	E-W	exposed	29	4 (14%)
AG	11 May 15	1030–1330	D	1.6	N	E-W	exposed	0	n/a
AG	7 May 15	1000–1330	D	1.6	N	E-W	exposed	0	n/a
AG	12 May 15	1100–1300	N	1.6	N	E-W	exposed	20	4 (20%)
ELG	5 May 15	0900–1600	D	6.4	N	E-W	shaded	68	58 (85%)
MG	9 May 15	1100–1300	D	0.3	W	N-S	exposed	0	n/a
MG	6 May 15	1000–1400	D	0.3	W	N-S	exposed	0	n/a
MG	8 May 15	1000–1400	D	0.3	W	N-S	exposed	0	n/a

Our VES surveys also revealed that diurnally-active toads were significantly more likely to flee upon approach than were nocturnally-active toads (X^2^ = 243.4, df = 1, p < 0.0001). On average, 89% (N = 161 observations) of diurnally-active toads fled upon our approach, compared to 13% (N = 274) of nocturnal toads (Table 2).

Cane toads fed during the day at both sites sampled. At Emma Gorge Plunge Pool, 11 of 15 (73%) dissected toad stomachs contained prey items, while at Ghost Bat Gorge 14 of 20 (70%) stomachs contained prey items. The full diversity of prey items was not quantified due to low sample sizes, but the Emma Gorge Plunge Pool samples were dominated by ants, and included wasps, and the Ghost Bat Gorge samples included ants, spiders, and beetles.

## Discussion

Cane toads in our study phase shifted from nocturnal to diurnal activity at some sites, a rarely demonstrated phenomenon in animals, especially at such a localized scale. A diversity of physical attributes of gorges within close proximity of one another provided a compelling case for environment (deeply shaded habitat) underpinning the shift. Timing of daily activity was consistent with solar exposure, which in turn was influenced by the orientation of the gorge axis and the width of the gorge or gorge section. Diurnally-active cane toads foraged during the day, and were more likely to flee upon approach. The shift to diurnal activity in some populations in our study likely reflects behavioral plasticity; cane toads arrived at our sites too recently to have evolved any genetic-based differences in activity. Collectively, our study suggests that a novel environment released cane toads from the constraint of nocturnal activity, allowing a phase shift in activity, followed by a shift in antipredator behavior. Cane toads may prefer to be diurnal, but more research is needed to test this idea, and to determine if diurnal activity is more beneficial than nocturnal activity, at some sites.

**Figure 5:**
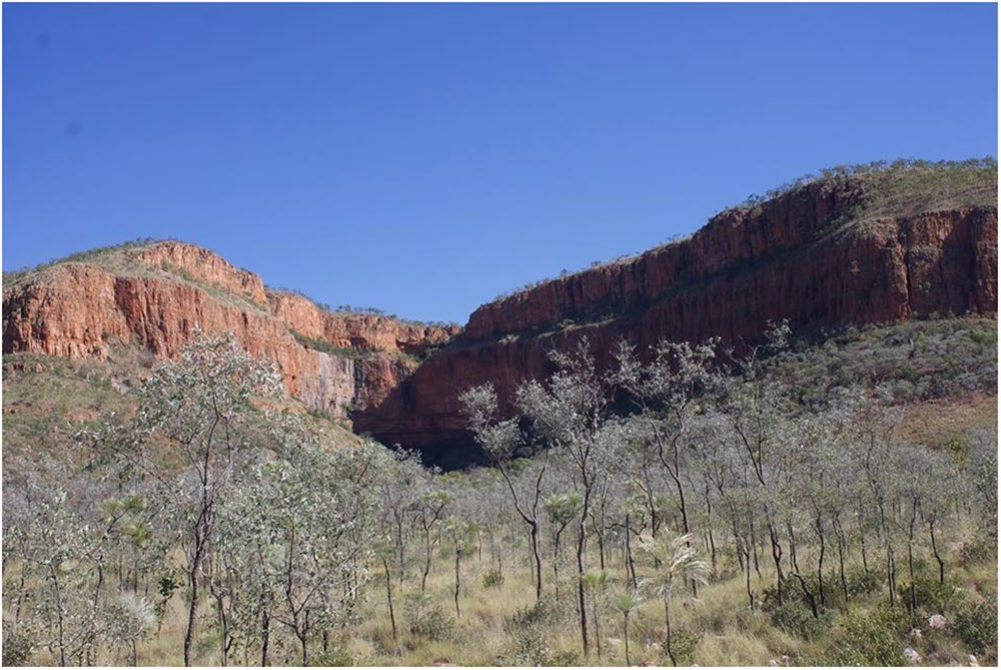
View from the east of Ghost Bat Gorge, where daily activity times of *R. marina* were primarily diurnal. The northern shadow provided deep shade that facilitated diurnal activity. Photograph was taken at 1030 hrs in June 2015.

Cane toads at Ghost Bat Gorge were predominantly diurnal, with activity peaking at 1000–1600 hrs for 288 toads at the two sites (Fig. 3b). Although the gorge is wide, it’s east-west orientation results in a deep northern shadow that shaded the camera sites (Fig. 5). In addition, VES surveys revealed diurnal activity in shade at El Questro Gorge, and at the Emma Gorge Plunge Pool (Fig. 3). The Emma Gorge Plunge Pool is a narrow section of gorge with a waterfall, springs, a large permanent waterhole, and rainforest elements. It receives direct sunlight for only about 2–3 hours per day. El Questro Gorge is a narrow (e.g., 30 m wide), deeply shaded gorge with a palm tree canopy and rainforest elements. Diurnal activity at these three gorges was in stark contrast to three sites at Emma Gorge in which activity was almost exclusively nocturnal, peaking at 1800–2000 hrs, and with <15 of 907 observations occurring during 0600–1800 hrs (Fig. 3). Toads were also nocturnal in Emma Gorge, Amalia Gorge and Moonshine Gorge (Tables 1, 2). These gorges are wide and exposed; Emma Gorge and Moonshine Gorge run north-south, and although Amalia Gorge runs east-west, it is more open than many of the other gorges, and the survey there was not in the northern shadow (as were the cameras in Ghost Bat Gorge, Fig. 5). A dichotomy of timing of activity (diurnal vs. nocturnal) among animal populations itself is not uncommon; populations of wide-ranging ectotherms are often diurnal in colder climates and nocturnal in hotter climates (e.g., toads:^24^; snakes:^25^), and populations can switch with a change in seasons (e.g., fish:^26^). However, in our study the dichotomy in timing of daily activity among sites occurred at a scale too fine to be explained by influences of overall climate. For example, Ghost Bat Gorge (diurnal toads) and Emma Gorge (nocturnal toads) are only ~7 km apart, and El Questro Gorge (diurnal toads) and Amalia Gorge (nocturnal toads) are only ~4 km apart (all of the sites are at a very similar elevation). Our study instead suggests that microhabitat caused by deep shade directly affected timing of activity.

Although we present compelling evidence for solar exposure via gorge orientation and width as the cause of the phase shift in activity, we cannot rule out other possibilities such as inter-site variation in predation and competition. We documented predation on cane toads by four predator species, of which two were diurnal (Black Kite, *Milvus migrans*; Blue-winged Kookaburra, *Dacelo leachii*) and two were mainly nocturnal (Keelback Snake, *Tropidonophis mairii*; Water Rat, *Hydromys chrysogaster*); however, we have no information on the relative abundance of these species at the different sites. Another possibility is that toads phase shifted to take advantage of an abundance of diurnally active food (e.g., ants were found in the stomachs of toads at Emma Gorge Plunge Pool), or that inter- or intra specific competition for food at night was higher at some sites than others. For example, although activity was mainly diurnal at Ghost Bat Gorge, 22% of toads were active at night; large numbers of toads relative to the amount of available food may have caused some individual toads to shift to diurnal foraging to avoid competing with nocturnally-active toads. In an experiment, European minnows became increasingly diurnal as their nutritional reserves declined^27^. Diurnal cane toads in the present study may have similarly been in a negative energy balance. Although we do not have comparative body condition data to test this idea, diurnal toads did not appear malnourished relative to nocturnal toads, and the former moved away upon approach. Higher wariness in diurnally-active toads compared to nocturnally-active toads may have been due to higher predation risk at night or higher conspicuousness to predators during the day. Or, toads may have been better able to detect potential predators during the day. A final possibility relates to condition; juvenile salmon in poorer condition were more likely to be diurnal despite the risk of predation^13^. Perhaps diurnal toads in our study were in poorer condition (we did not measure it).

Diurnal activity also contrasted with the vast majority of studies and observations of cane toad activity worldwide (Table 3). Of 22 studies or observations spanning 12 countries, 19 described cane toads as nocturnal. Exceptions were one individual toad active in low undergrowth in a swampy area in Palau^28^, another individual rehydrating on a moist rotten log in Puerto Rico^29^, and mass numbers of individuals rehydrating in reservoirs in Australia^23^ (see below). The claim of a preponderance of diurnal activity in cane toads in Panama^30^ is thought to be in error; according to Zug and Zug^31^, toads in that study may have actually been *Bufo typhonius*, an exclusively diurnal species that is sympatric with cane toads in Panama. The one definite case of diurnal activity in cane toads is the study by Webb *et al*.^23^, who demonstrated that toads rehydrated in reservoirs during the day in an arid zone population. The authors concluded that this behavioral flexibility may have facilitated the spread of toads into drier, hotter regions than those experienced in its native range by reducing exposure to desiccation and thermal stress suffered during the day in terrestrial sheltering sites. However, it is difficult to envision diurnal activity in our study performing a similar function.

**Table 3:** Summary of activity (nocturnal vs. diurnal) in adult *R. marina* from populations around the world. N = native, I = invasive.

Location	N/I	Activity	Comment	Source
American Samoa	I	nocturnal	Toads were collected 2–4 hours after sunset to allow foraging	Grant, 1996
Australia	I	Diurnal	Rehydrated in dams during the day	Webb *et al*., 2014
Australia	I	Both	Active by day at some sites and at night at other sites	Present study
Australia	I	nocturnal	90% nocturnal	Freeland & Kerin, 1991
Australia	I	nocturnal	Toads emerged at night and sheltered during the day	Seebacher & Alford 1999
Bermuda	I	nocturnal	Large numbers killed by cars at night	Wingate, 1965
Costa Rica	N	nocturnal	(cane) toads are nocturnal	Savage, 2005
Costa Rica	N	nocturnal	Active at night, shelter during the day	Zug, 1983
general	—	nocturnal	Cane toads are mainly nocturnal animals	Lever, 2001
Guadeloupe	I	(nocturnal)	Shelter during the day in caves, under limestone outcrops and fallen tree trunks, in road drains and gutters and beneath bridges	M. Breuil, pers. comm. In Lever, 2001
Hawaii	I	Both, mainly nocturnal	Toads feed anytime during day or night, but are most active after sunset	Pemberton, 1934
Hawaii	I	nocturnal	Low numbers of ‘nocturnal’ toads in stomachs of diurnal mongoose	Baldwin *et al*., 1952
Hawaii	I	nocturnal	Ubiquitous during the hours of darkness on roads	Wright, 1992
Montserrat	I	nocturnal	Cane toads forage almost exclusively at night	Blankenship, 1990
Palau	I	Diurnal	One individual active by day in low undergrowth near a swampy area	Crombie & Pregill, 1999
Panama	N	nocturnal	Toads feeding under lights at night	Brattstrom 1962
Panama	N	nocturnal	(cane) toads are predominantly nocturnal	Zug & Zug, 1979
Panama	N	Diurnal	preponderant activity was diurnal	Park *et al*., 1940
Philippines	I	(nocturnal)	Diurnal sheltering	Rabor, 1952
Puerto Rico	I	nocturnal	Toads hide during the day but appear in numbers at night	Grant, 1931
Puerto Rico	I	nocturnal	Movement to and from activity centers and rehydration sites at night	Carpenter & Gillingham, 1987
Puerto Rico	I	Diurnal	One individual rehydrated on a moist rotten log at midday	Carpenter & Gillingham, 1987
USA	I	Both	Nocturnal, except for a few instances of diurnal feeding and calling	Krakauer, 1968

Cane toads have dispersed steadily westward across northern Australia since their introduction in 1935. Thus, while toads arriving in our study areas had not experienced gorge habitats, their ancestral populations did encounter a few gorges as toads dispersed across northern Australia. However, the vast majority of habitat encountered over their 80-year colonization would have been flat open woodland in the tropical savannah ecosystem. Exposure to full sun in this habitat would generally preclude diurnal activity due to the rarity of deep shade. In contrast, the Kimberley region encompassing our study areas is gorge-rich; sandstone gorges, many with perennial water, dissect much of the rocky landscape. Deeply shaded habitats appear to have released some toads from obligatory nocturnal behavior. Do cane toads prefer to be diurnal? Our data are not sufficient to answer this intriguing question, nor can we determine if diurnal activity was beneficial relative to nocturnal activity without further research.

There were a few limitations to our study. Our study design was not ideal; we had little replication at the gorge scale, and cameras were employed to test other hypotheses in a concurrent study. Our camera data also did not allow us to distinguish between individual toads; thus, at sites with smaller sample sizes (e.g., Saddleback) the number of observations may have reflected fewer toads repeatedly moving past the cameras. Finally, the lack of wet season activity data from Emma Gorge prevents us from building a more complete picture of activity in that population. Nevertheless, our study clearly documented a phase shift in activity, including feeding, in a population of cane toads near the invasion front. Nocturnal activity in nearby populations and elsewhere suggests that the diurnal activity reflects recent behavioral plasticity. Although it is currently unclear if diurnal activity in our study achieves benefits relative to nocturnal activity, such plasticity can promote successful species invasions^16,32,33^. Indeed, behavioral plasticity in cane toads, in general, may help explain their successful colonization^17,23^. Regardless, the apparent plasticity we revealed in the current study may mean more bad news for managers attempting to control cane toads.

## Data Availability

The datasets generated and analysed during the current study are available from the corresponding author on reasonable request.
